# Differential recognition of lipid domains by two Gb3-binding lectins

**DOI:** 10.1038/s41598-020-66522-8

**Published:** 2020-06-16

**Authors:** Thomas Schubert, Taras Sych, Josef Madl, Maokai Xu, Ramin Omidvar, Lukas J. Patalag, Annika Ries, Katharina Kettelhoit, Annette Brandel, Yves Mely, Claudia Steinem, Daniel B. Werz, Roland Thuenauer, Winfried Römer

**Affiliations:** 1 Faculty of Biology, Albert-Ludwigs-University Freiburg, Freiburg, Germany; 2 Synthetic Biology of Signalling Processes, Signalling Research Centres BIOSS and CIBSS, Albert-Ludwigs-University Freiburg, Freiburg, Germany; 3 Toolbox, BIOSS Centre for Biological Signalling Studies, Albert-Ludwigs-University Freiburg, Freiburg, Germany; 4 Laboratory of Bioimaging and Pathologies, UMR 7021 CNRS, Faculty of Pharmacy, University of Strasbourg, Strasbourg, France; 5 Technische Universität Braunschweig, Institut für Organische Chemie, Braunschweig, Germany; 6 Institut für Organische und Biomolekulare Chemie, Georg-August-Universität Göttingen, Göttingen, Germany; 7 Advanced Light and Fluorescence Microscopy Facility, Centre for Structural Systems Biology (CSSB) and University of Hamburg, Hamburg, Germany; 8 Present Address: Institute for Experimental Cardiovascular Medicine, University Heart Center Freiburg - Bad Krozingen and Faculty of Medicine, University of Freiburg, Freiburg, Germany

**Keywords:** Sensors and probes, Membrane biophysics, Cellular imaging

## Abstract

The two lectins LecA from *Pseudomonas aeruginosa* and the B-subunit of Shiga toxin from *Shigella dysenteriae* (StxB) share the glycosphingolipid globotriaosylceramide (Gb3) as receptor. Counterintuitively, we found that LecA and StxB segregated into different domains after recognizing Gb3 at the plasma membrane of cells. We hypothesized that the orientation of the carbohydrate head group of Gb3 embedded in the lipid bilayer differentially influences LecA and StxB binding. To test this hypothesis, we reconstituted lectin-Gb3 interaction using giant unilamellar vesicles and were indeed able to rebuild LecA and StxB segregation. Both, the Gb3 fatty acyl chain structure and the local membrane environment, modulated Gb3 recognition by LecA and StxB. Specifically, StxB preferred more ordered membranes compared to LecA. Based on our findings, we propose comparing staining patterns of LecA and StxB as an alternative method to assess membrane order in cells. To verify this approach, we re-established that the apical plasma membrane of epithelial cells is more ordered than the basolateral plasma membrane. Additionally, we found that StxB recognized Gb3 at the primary cilium and the periciliary membrane, whereas LecA only bound periciliary Gb3. This suggests that the ciliary membrane is of higher order than the surrounding periciliary membrane.

## Introduction

The plasma membrane controls the movement of substances between the interior and the outside of a cell. It is based on a lipid bilayer matrix that consists of lipids with diverse head groups and fatty acyl chains. The different membrane lipids are inhomogeneously distributed in the plasma membrane: First, the inner and the outer leaflets of the lipid bilayer are composed of different lipid classes and this asymmetry is actively maintained by the cell^1–4^. Second, the plasma membrane is also heterogeneous along each leaflet: lipid domains of specific composition are formed^5–7^ that serve as platforms for various cellular processes such as endocytosis^8^ and signal transduction^9–11^ and manifest in locally altered membrane order^12–14^.

Detecting and following lipid domains in cells has turned out to be a challenging endeavor^15,16^. Direct fluorescent labeling of a lipid is casting doubt on its natural behavior and phase partitioning properties^17–19^. As alternative, environmentally sensitive probes that intercalate in the lipid bilayer and change their fluorescence spectra dependent on membrane order have been extensively used^19^. However, such probes often show cytotoxic effects, can require technically advanced equipment, and can cause side effects by altering membrane order^20^. An alternative approach is based on carbohydrate-binding proteins, so called lectins, which specifically recognize the carbohydrate-moieties of glycosphingolipids^21^. For example, the B-subunit of cholera toxin (CtxB) targets the ganglioside monosialotetrahexosylganglioside (GM1). It has become a frequently used marker for highly ordered membrane domains termed as ‘lipid rafts’^22–25^. Additionally, non-toxic derivatives of lipid-binding pore-forming toxins can be used as membrane domain markers^26^.

Here, we report that the lectins LecA from *Pseudomonas aeruginosa*^27–29^ and the B-subunit of Shiga toxin (StxB) from *Shigella dysenteriae*^30–32^, which recognize the same glycosphingolipid, the globotriaosylceramide (Gb3, also referred to as P^K^ blood group antigen and CD77^33^), segregate into different domains at the plasma membrane of cells. Furthermore, we were able to successfully rebuild LecA and StxB spatial segregation in synthetic lipid bilayer systems - giant unilamellar vesicles (GUVs) and supported lipid bilayers (SLBs). Such artificially prepared membranes with controlled lipid composition are extensively employed for studying lipid organization and function^14,34,35^. They allow to choose conditions to achieve lateral lipid segregation^5,36,37^, resulting in the formation of liquid disordered (ld) and liquid ordered (lo) phases, which resembles lateral cellular plasma membrane heterogeneities. Experiments with GUVs and SLBs enabled us to identify the interplay between the molecular structure of Gb3 (i.e. which Gb3 species it is) and the membrane environment, which both determine Gb3 membrane embedding and head group orientation^38^, as critical parameters causing differential Gb3 recognition by LecA and StxB. In particular, we revealed that StxB has a preference for more ordered domains in comparison to LecA.

Based on these results we suggest that comparing the staining patterns of LecA and StxB yields a novel approach for evaluating membrane order in cells. To demonstrate the feasibility of this approach, we used epithelial cells. The apical plasma membranes of epithelial cells are known to be more ordered than basolateral plasma membranes^2^. As expected from this order difference, StxB showed a stronger preference for the apical plasma membrane in comparison to LecA. In addition, we found that StxB, but not LecA, was able to recognize the primary cilium. This surprising finding suggests that the membrane of the primary cilium contains a lipid domain that has a higher order than the surrounding apical plasma membrane.

## Results

### LecA and StxB recognize different lipid domains on cells

**Figure 1:**
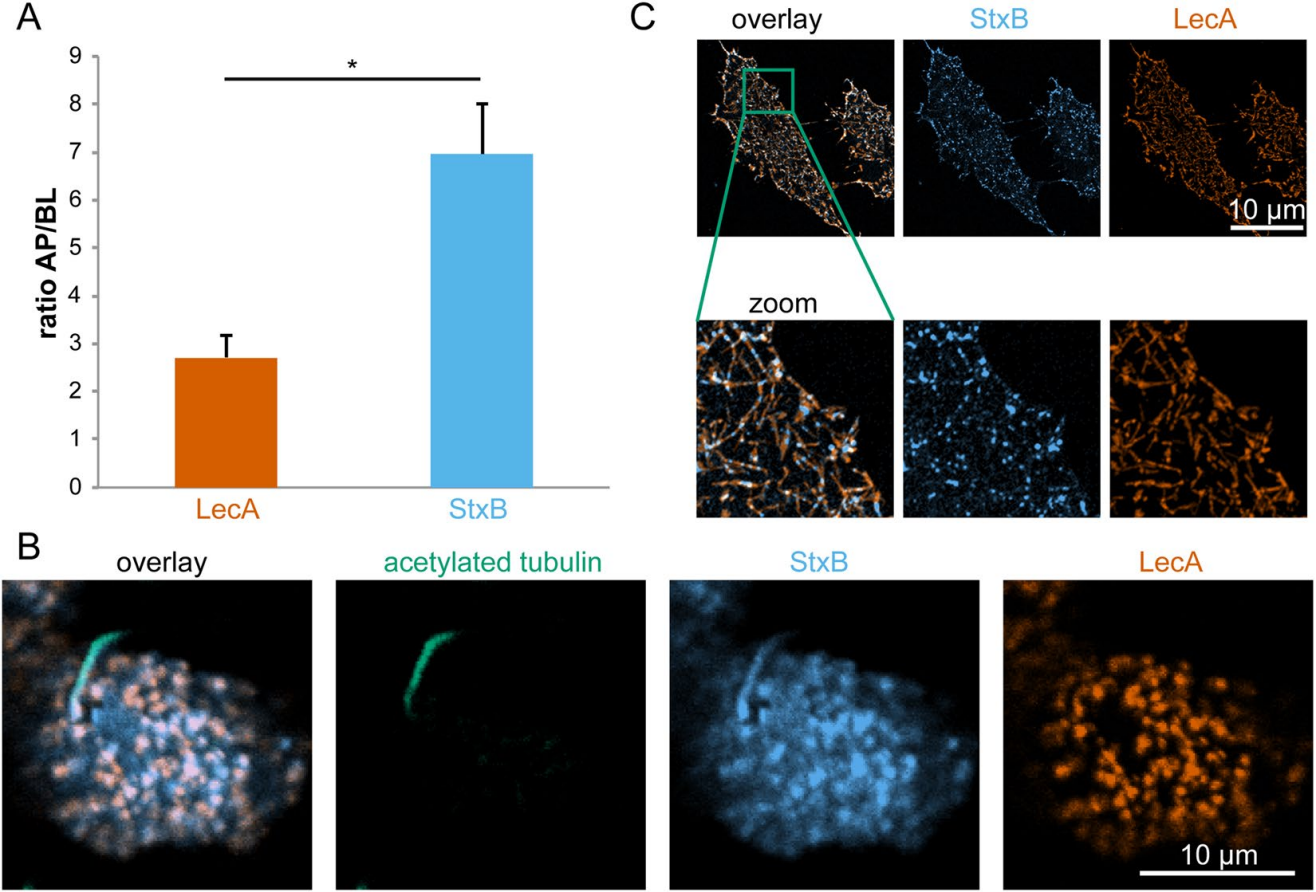
LecA and StxB show different binding patterns on cells. (**A**) MDCK cells stably expressing Gb3 synthase were mixed with wild type MDCK cells 1:10, seeded on transwell filters, and cultured for 4 d to form polarized monolayers. LecA-Alexa488 (117 nM) or StxB-Cy3 (106 nM) were applied only to the apical (AP) or basolateral (BL) side of the cells and incubated for 30 min at 4 °C. After washout, samples were fixed, mounted, and the intensities of AP or BL bound LecA or StxB were determined in single cells by measuring LecA-Alexa488 and StxB-Cy3 intensities from image stacks recorded with a confocal microscope. Then, the AP to BL signal intensities were calculated from the mean values from n > 50 cells per condition. The graphs display the mean values from 3 independent experiments, the error bars represent the standard error mean. Statistical significance was assayed with a paired two-tailed t-test, * indicates p < 0.05. (**B**) Polarized MDCK cells stably expressing Gb3 synthase were incubated apically with LecA-Alexa488 (196 nM, orange) and StxB-Cy3 (13 nM, blue) for 30 min at 37 °C. After washout of unbound lectin, cells were fixed and primary cilia were stained using an antibody recognizing acetylated tubulin (green). The images show confocal sections at the height of the apical plasma membrane. (**C**) HeLa cells were energy-depleted and then incubated with LecA-Alexa488 (98 nM) or StxB-Cy3 (106 nM) for 15 min at 37 °C. After fixation, cells were imaged with a TIRF-SIM microscope.

The two lectins LecA and StxB share the same receptor, the globoside Gb3^31,39^. Upon application of LecA and StxB to either the apical or basolateral side of polarized Madin-Darby canine kidney (MDCK) strain II cells, stably expressing Gb3 synthase^9^, both lectins were found at higher concentration at the apical cell membranes than at the basolateral ones (Fig. 1A). However, StxB showed a significantly stronger preference for the apical plasma membrane than LecA (apical to basolateral signal intensity ratio for StxB 7 ± 1 versus 2.7 ± 0.5 for LecA). Furthermore, we found that StxB was able to bind to the primary cilium (Fig. 1B), which confirms that Gb3 lipids are also found in this cellular organelle that shares a continuous lipid bilayer with the apical plasma membrane. Strikingly however, no LecA binding was detected at the cilium, even at relatively high LecA concentrations (Fig. 1B). In control experiments we verified that the selective binding of StxB to the primary cilium was not due to a potential interference between StxB and LecA, because we found the same binding behavior also in experiments in which only StxB or LecA was applied (Fig. S1). Also, the reported hetero-multivalency of LecA^40^ does not play a role here since in Gb3-negative MDCK cells no binding of either LecA or StxB was observed (Fig. S1). Since the cilium represents only a small fraction of the area of the apical plasma membrane^41^, StxB binding to the primary cilium cannot fully explain the higher apical to basolateral binding ratio observed for StxB in comparison to LecA. Measuring the total amount of bound lectin at the apical and basolateral plasma membrane neglects a potential segregation of the two lectins at the plasma membrane on a smaller scale. Indeed, Fig. 1B shows that at the (non-ciliary) apical plasma membrane LecA and StxB displayed only partially overlapping binding patterns. In agreement, previous studies found a partial segregation of the two lectins during early stages of their endocytosis^9^, thus suggesting that the segregation between LecA and StxB occurs already at the plasma membrane.

To independently confirm these findings, we used HeLa cells, which endogenously express Gb3^42^. We applied energy-depletion in order to inhibit energy-dependent scission of endocytic buds, which results in the formation of fairly stable tubular invaginations at the plasma membrane^8^. The super-resolution microscopy technique TIRF-SIM revealed that LecA and StxB clearly segregate into different domains at tubular invaginations from the plasma membrane (Fig. 1C).

These findings demonstrate that - despite sharing the same receptor - LecA and StxB show different binding patterns on cells.

### LecA and StxB also segregate on GUVs containing a mixture of Gb3 species

Various model systems have been previously used to study the effects of StxB on different membranes^8,12,14^. We have recently also employed an artificial membrane system to understand the impact of LecA on synthetic protocells and the ability to form multivalent interactions^43^.

**Figure 2:**
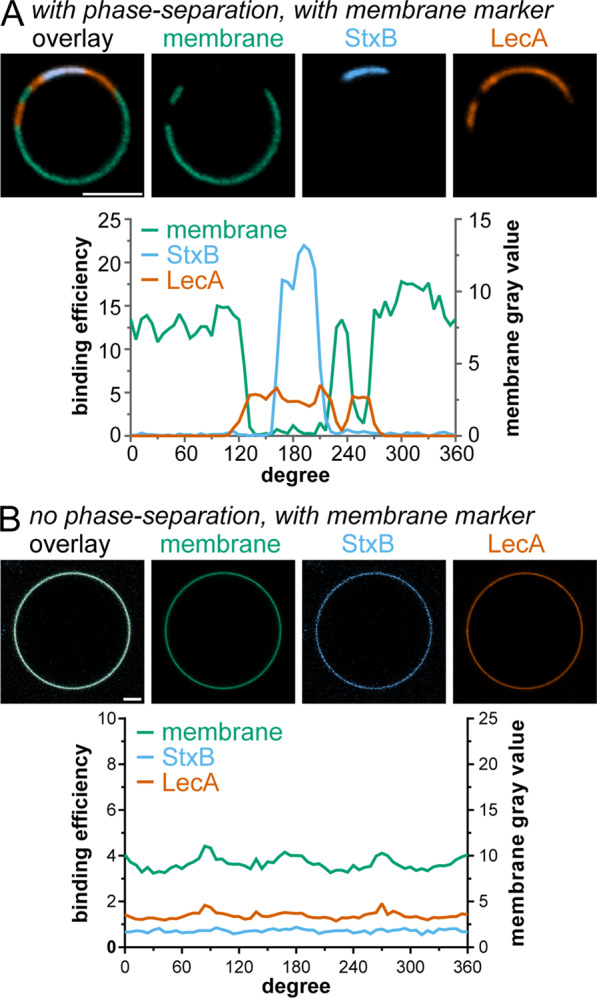
Segregation between LecA and StxB can be reconstituted in GUVs containing a natural mixture of Gb3 species. GUVs containing 5 mol-% Gb3-mix were incubated with LecA-Al647 (200 nM, orange) and StxB-Cy3 (600 nM in A and 200 nM in B, blue) (**A,B**). The images show equatorial sections through representative GUVs with a diagram displaying the corresponding circular intensity profiles underneath. Phase-separated GUV (**A**) contained DOPC/cholesterol/sphingomyelin/Gb3-mix in the ratio 42.5/14.5/42.5/5 mol-%, whereas non-phase-separated GUV (**B**) contained DOPC/cholesterol/Gb3-mix in the ratio 64.5/30/5 mol-%. GUVs in (**A,B**) were additionally spiked with 0.5 mol-% of the membrane marker β-BODIPY-FL-C_5_-HPC (green). Scale bars correspond to 5 μm.

In order to identify the mechanism causing LecA and StxB spatial segregation, we applied GUVs with embedded Gb3 as membrane model system. We produced GUVs containing 5 mol-% of a natural mixture of Gb3 species (Gb3-mix) and applied both lectins (Fig. 2). Under certain conditions, StxB can induce tubular invaginations on GUVs^8,44^. Hence, to rule out secondary effects, we started imaging after lectin addition and only analyzed images from time points before tubular invaginations formed. LecA and StxB showed spatially separated binding patterns on the surface of lo/ld-phase-separated GUVs (Fig. 2A). The ld phase was visualized by including the ld phase marker β-BODIPY-FL-C_5_-HPC^17^. The fluorescence micrographs revealed that both lectins preferred the lo phase, but LecA and StxB only partially overlapped within the lo phase. In contrast, in non-phase-separated GUVs no segregation between LecA and StxB was observed (Fig. 2B).

To rule out non-specific effects, we carried out several control experiments. First, we established that GUVs lacking Gb3 were not able to bind LecA or StxB (Fig. S2). Second, only negligible spectral crosstalk occurred between the membrane marker channel (β-BODIPY-FL-C_5_-HPC) and the channels used for the fluorescent labels of the lectins (Cy3 and Cy5) (Fig. S3).

These results demonstrate that the different binding patterns between LecA and StxB, which were observed in cells, could be successfully rebuilt in a model system of controlled composition based on GUVs. In such synthetic systems, in absence of the active cellular machinery, only the lectin-Gb3 interaction and the membrane environment can drive the spatial segregation between LecA and StxB.

### Analysis of LecA and StxB preference for Gb3 species and membrane order

Based on previously reported observations with a specific class of Gb3-binding lectins, pentameric vero toxins^45–50^, we hypothesized that LecA and StxB prefer to recognize differently exposed carbohydrate head groups of Gb3. The exposure of the head group of a glycolipid is predominantly determined by the way it is embedded in the lipid bilayer^49,51^. The embedding itself is influenced by (i) the length and saturation degree of its fatty acyl chain and (ii) the constitution of the surrounding lipid environment. To test how the structure of the fatty acyl chains influences LecA and StxB binding, we produced GUVs containing only Gb3 molecules with defined fatty acyl chains (Gb3-C24:0, Gb3-C24:1, and Gb3-FSL) and Gb3-mix as a control. Hereby, Gb3-FSL is a synthetic lipid that contains the Gb3 head group linked via a spacer (O(CH_2_)_3_NH) to a dioleoylphosphatidylethanolamine (DOPE) backbone^52^. Furthermore, to investigate how the lipid environment influences LecA and StxB binding, we prepared non-phase-separated and phase-separated GUVs. To measure the binding of the two lectins in a quantitative manner, we used the recently developed FIJI macro GUV-AP that determines protein binding efficiency to GUVs and enables analysis of large datasets^53^. For quantifying the binding efficiency, we determined the ratio between the signal of the lectin at the rim of the GUV and free lectin in solution^53^. Such ratio represents quantitatively the contrast between bound and free lectin. If the lectin cannot bind to the GUV, the contrast and thus the binding efficiency equals to 0. Furthermore, we included a feature in the macro to discriminate protein binding to ld and lo phases in phase-separated GUVs.

**Figure 3:**
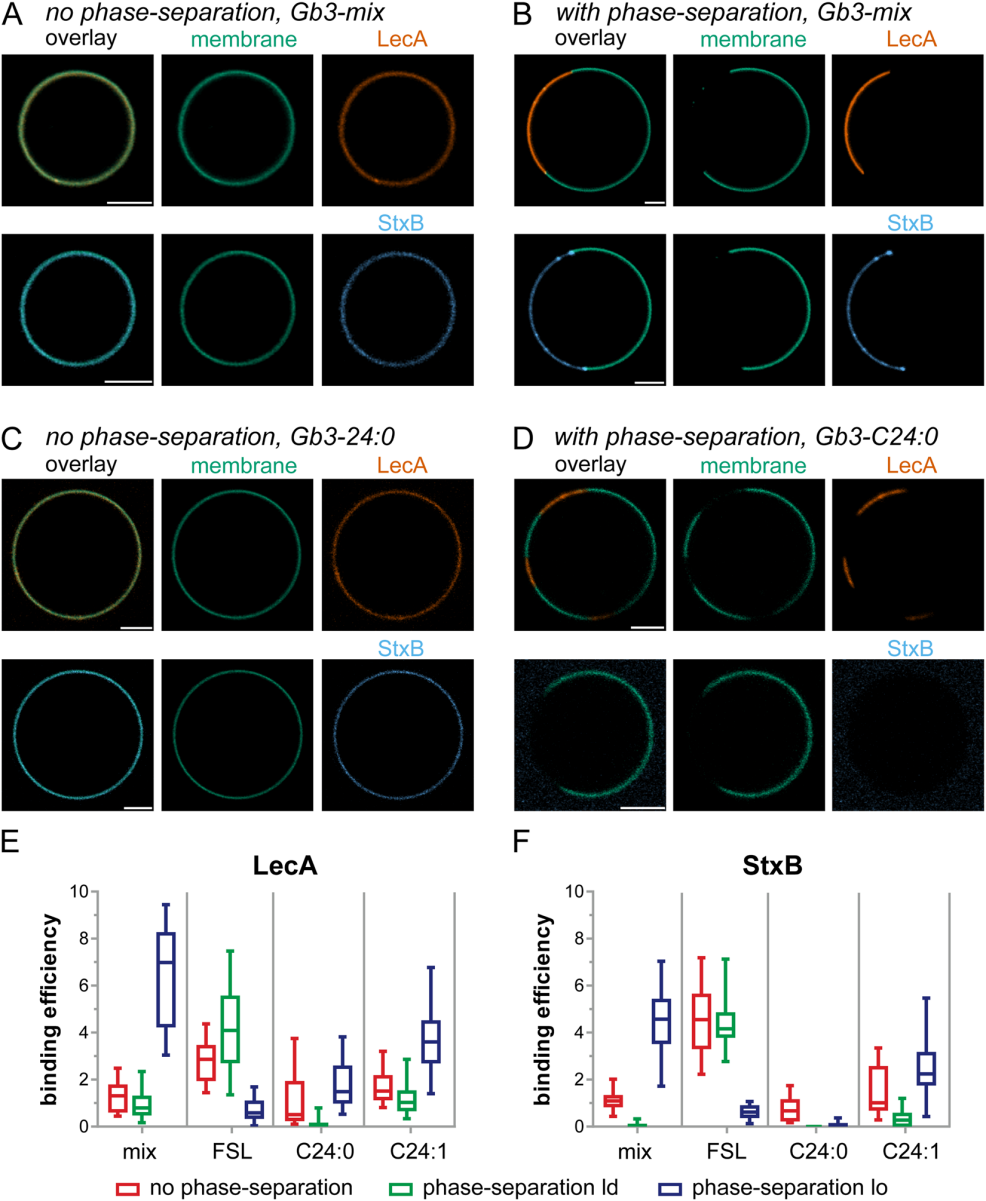
Investigations how Gb3 species and phase separation influence LecA and StxB binding efficiencies. GUVs doped with 0.5 mol-% of the membrane marker β-BODIPY-FL-C_5_-HPC (green) and 5 mol-% Gb3-mix (**A,B**) or 5 mol-% Gb3-C24:0 (**C,D**) were incubated with LecA-Cy5 (200 nM, orange) or StxB-Cy5 (200 nM, blue). Non-phase-separated GUVs ((**A,C**) contained DOPC/cholesterol/Gb3 in the ratio 64.5/30/5 mol-%, whereas phase-separated GUVs (**B,D**) consisted of DOPC/cholesterol/sphingomyelin/Gb3 in the ratio 42.5/14.5/42.5/5 mol-%. The images A – D show equatorial sections through representative GUVs. Scale bars correspond to 5 μm. (**E,F**) Quantitative analysis of the binding efficiencies of LecA (**E**) and StxB (**F**) to non-phase-separated GUVs (red) or the ld (green) or lo (blue) phase of phase-separated GUVs containing 5 mol-% of either Gb3-mix (mix), Gb3-C24:0 (C24:0), Gb3-C24:1 (C24:1), or Gb3-FSL (FSL). For each condition, the middle horizontal line represents the median, the boxes the 25^th^ to 75^th^ percentiles, and the whiskers the min and max values. In Table S1 the descriptive statistics of the data are summarized and Table S2 contains the data from a significance analysis.

In non-phase-separated GUVs containing Gb3-mix, separately applied LecA and StxB were able to bind homogenously (Fig. 3A) and with comparable binding efficiency (Fig. 3E,F and Table S1,S2). Introducing phase-separation to GUVs containing Gb3-mix indicated that both, LecA and StxB, preferentially bound lo phases (Fig. 3B) as it has already been shown before (Fig. 2A). Interestingly, LecA recognized Gb3-mix in the lo phase better (binding efficiency 7 ± 2) than StxB (binding efficiency 4.5 ± 1.4).

In non-phase-separated GUVs containing Gb3-C24:0, LecA and StxB were able to bind with similar efficiency (Fig. 3C). But in phase-separated GUVs containing Gb3-C24:0, clear differences became apparent: Only LecA detectably bound to lo phases, whereas StxB hardly bound lo phases (Fig. 3D–F). In GUVs containing Gb3-C24:1 both lectins exhibited similar binding patterns (Fig. S4A and Fig. 3E,F).

In general, for Gb3-mix and all single Gb3 species tested so far, both lectins preferred the lo phase over the ld phase in phase-separated GUVs. However, Gb3-FSL demonstrated the opposite behavior: In phase-separated GUVs containing Gb3-FSL, LecA and StxB preferred the ld phase over the lo phase (Fig. S4B and Fig. 3E,F). In addition, whereas LecA displayed higher binding efficiencies than StxB in non-phase-separated GUVs containing Gb3-C24:0 and Gb3-C24:1, the opposite was true for non-phase-separated GUVs containing Gb3-FSL (Fig. S4B and Fig. 3E,F).

These results demonstrate that by altering the incorporation of Gb3 in the lipid bilayer, different binding behavior of LecA *versus* StxB can be achieved.

**Figure 4:**
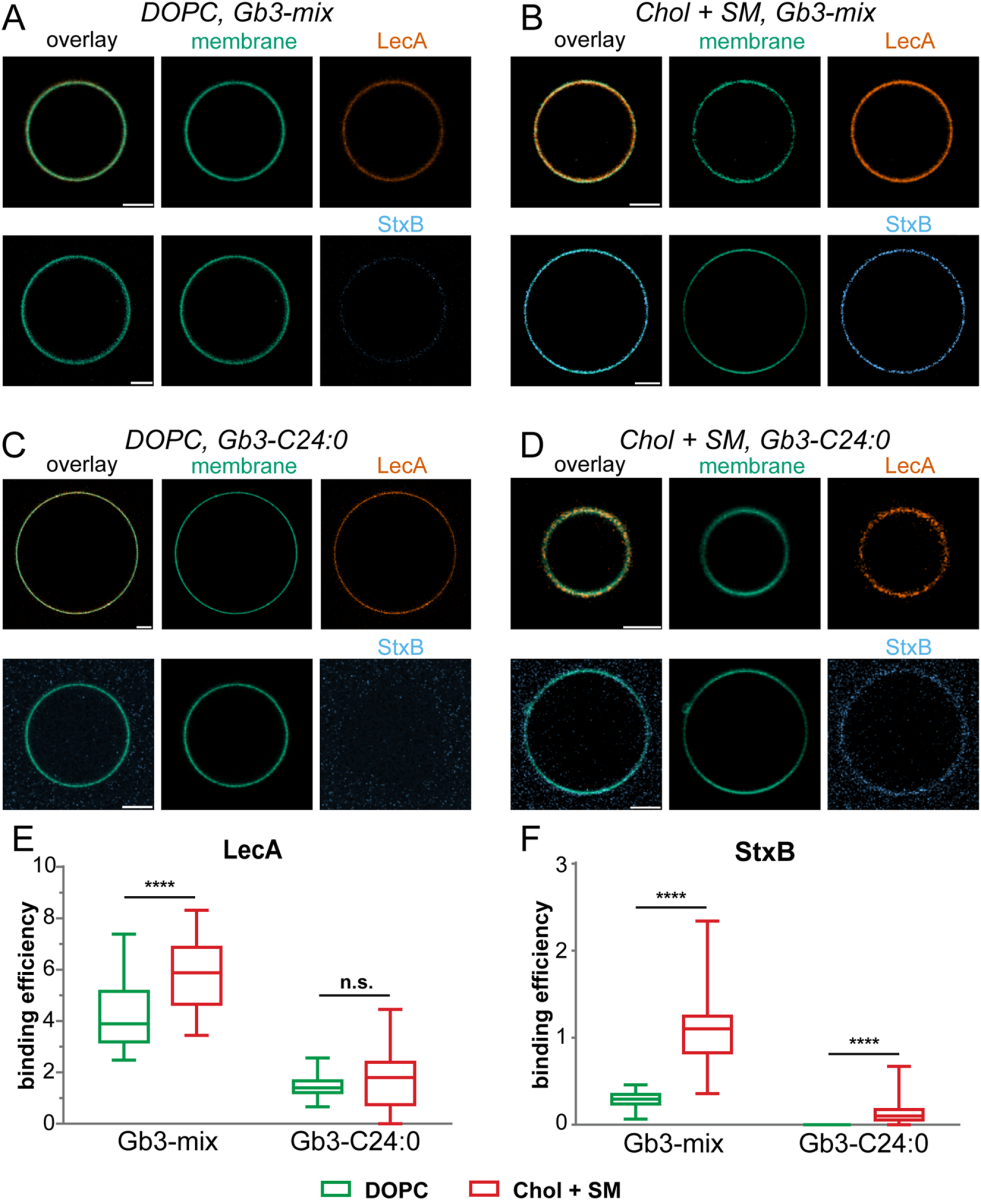
The interplay between Gb3 species and cholesterol content influences LecA and StxB binding efficiencies. GUVs doped with 0.5 mol-% of the membrane marker β-BODIPY-FL-C_5_-HPC (green) and 5 mol-% Gb3-mix (**A,B**) or 5 mol-% Gb3-C24:0 (**C,D**) were incubated with LecA-Al647 (200 nM, orange) or StxB-Cy5 (200 nM, blue). GUVs approximating liquid-disordered membranes (DOPC, A and C) contained DOPC/Gb3 in the ratio 94.5/5 mol-%, and GUVs resembling liquid-ordered membranes (Chol + SM, **B,D**) consisted of sphingomyelin/cholesterol/Gb3 in the ratio 64.5/30/5 mol-%. The images (**A–D**) show equatorial sections through representative GUVs. Scale bars correspond to 5 μm. (**E,F**) Quantitative analysis of the binding efficiencies of LecA (**E**) and StxB (**F**) to DOPC GUVs (green) and Chol + SM GUVs (red) containing 5 mol-% of either Gb3-mix, or Gb3-C24:0. For each condition, the middle horizontal line represents the median, the boxes the 25^th^ to 75^th^ percentiles, and the whiskers the min and max values. In Table S3 the descriptive statistics of the data are summarized, and Table S4 contains the complete data from the significance analysis.

The most obvious difference was seen for Gb3-C24:0, where LecA was able to recognize lo phase-localized Gb3, but StxB hardly bound. This difference could be caused by the lipid order of the lo phase domains that is dependent on cholesterol and sphingomyelin^5,36,54^. Thus, to test the impact of cholesterol and sphingomyelin directly, we prepared ‘pure phase’ GUVs that did not contain cholesterol and sphingomyelin and approximate ld phases (‘DOPC’ in Fig. 4) and GUVs that did contain cholesterol and sphingomyelin approximating lo phases (‘Chol + SM’ in Fig. 4). Chol + SM-GUVs bound both lectins significantly better than DOPC-GUVs (Fig. 4A,B,E,F), but the relative differences between Chol + SM-GUVs and DOPC-GUVs were more pronounced for StxB. This effect was enhanced for GUVs containing only Gb3-C24:0. In this case, LecA equally bound to DOPC-GUVs and Chol + SM-GUVs, but StxB was only able to recognize Chol + SM-GUVs (Fig. 4C–F and Table S3,S4).

Taken together, using GUVs as model system enabled us to reveal that the two parameters ‘Gb3 fatty acyl chain structure’ and ‘lipid environment’ can cause conditions leading to preferential binding of either LecA or StxB. Both parameters influence Gb3 embedding in the lipid bilayer, which determines the orientation of the Gb3 head group^45,49,51^. The head group conformation is known to affect the binding efficiency of lectins to glycosphingolipids^38^. Our analysis demonstrated that both parameters have to be considered to explain the different binding behavior of LecA and StxB.

### Redesign of LecA and StxB segregation using a minimal system with pure Gb3 species

**Figure 5:**
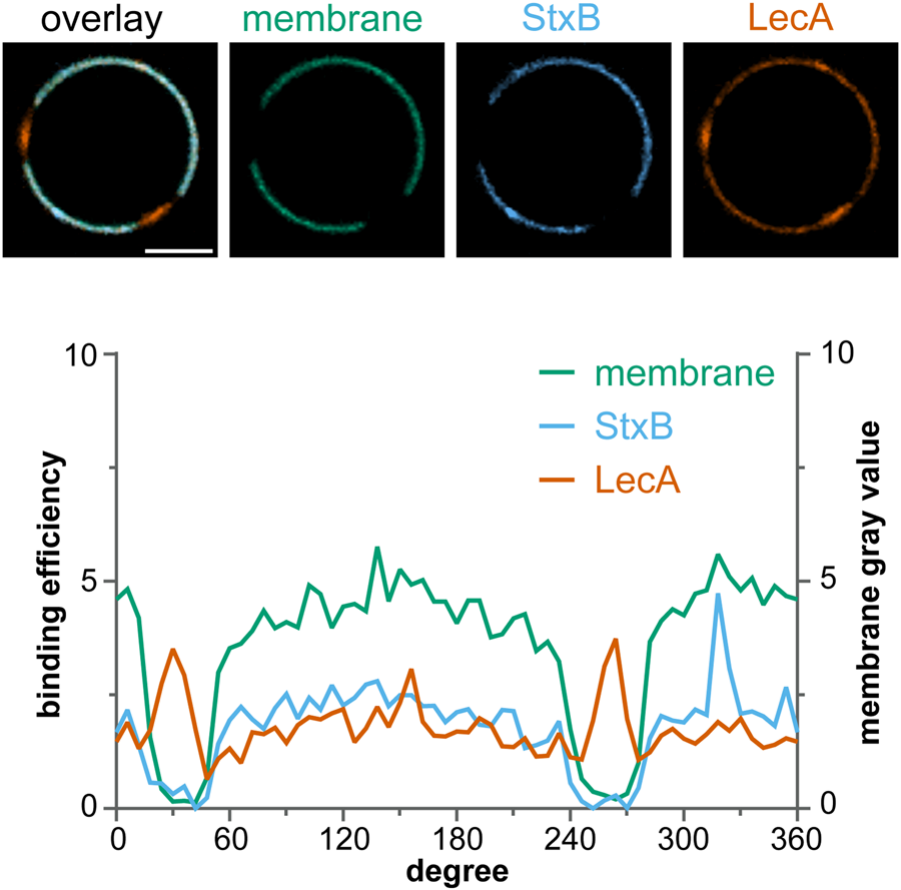
Reconstituting LecA and StxB segregation in minimal systems. Phase-separated GUVs composed of DOPC/cholesterol/sphingomyelin/β-BODIPY-FL-C_5_-HPC (green)/Gb3-FSL/Gb3-C24:0 in the ratio 40/14.5/40/0.5/5/5 mol-%) were incubated with LecA-Al647 (200 nM, orange) and StxB-Cy3 (600 nM, blue). Equatorial sections through a representative GUV are shown and the corresponding circular intensity profiles are displayed underneath. Scale bar corresponds to 5 μm.

If the hypothesis is correct that the combination of Gb3 fatty acyl chain structure and membrane environment differentially determines the binding preferences of LecA and StxB, it should be possible to rebuild LecA and StxB segregation on the same GUV using a minimal set of distinct Gb3 species. Since we observed the strongest differences between StxB and LecA for Gb3-C24:0 and Gb3-FSL, we chose to test LecA and StxB segregation in phase-separated GUVs containing 5 mol-% of each of these two Gb3 species. Indeed, this was sufficient to achieve different, but partially overlapping, staining patterns of LecA and StxB (Fig. 5) that resembled the patterns observed on cells (Fig. 1B,C) and on phase-separated GUVs containing Gb3 mix (Fig. 2A). In particular, in GUVs containing only Gb3-C24:0 and Gb3-FSL, StxB was only able to bind the ld phase, whereas LecA showed a preference for the lo phase.

Moreover, we investigated whether such segregation can also be observed in phase-separated SLBs containing 5 mol-% of Gb3-C24:0 and 5 mol-% of Gb3-FSL (Fig. S5A). Single microscopic images of flat SLBs provide a larger overview along the lateral direction of the lipid bilayer than images of GUVs. Therefore, in the images of SLBs multiple StxB clusters with distinct binding efficiencies for StxB within the ld phase became apparent. LecA bound in a pattern inverse to the StxB binding pattern and preferentially recognized lo phases. To gain more insights into LecA and StxB binding behavior on SLBs, we carried out experiments with SLBs containing only single Gb3 species. In SLBs containing Gb3-FSL, LecA formed clusters in the ld phase (Fig. S5B) and StxB homogeneously bound ld phases (Fig. S5C). SLBs containing only Gb3-C24:0 showed preferential binding of LecA to lo phases (Fig. S5D). Surprisingly, in these SLBs StxB also bound lo phases (Fig. S5E) as also previously reported^55^. However, this is in contrast to the results obtained with phase-separated GUVs containing Gb3-C24:0, where StxB was not able to bind significantly (Fig. 3D). This observation suggest that an additional parameter, such as altered inter-leaflet coupling in SLBs caused by the interaction of one leaflet with the underlying mica substrate, results in changes in Gb3 head group exposure that can be sensed by StxB.

Taken together, our results demonstrate that a minimal system containing only two selected Gb3 species is sufficient to replicate different binding patterns of LecA and StxB.

## Discussion

### The concept of LecA and StxB as sensors for membrane environment

LecA and StxB bind both to the glycosphingolipid Gb3^31,39^. However, we found that LecA and StxB spatially segregated at the plasma membrane of cells. By using GUVs, which are artificial membrane systems that mimic cellular plasma membranes and can be produced with well-defined lipid composition and phases, we were able to replicate the segregation between LecA and StxB. This proves that the different binding behavior of LecA and StxB is based on the direct interaction between the lectins and Gb3. Moreover, we identified two interdependent parameters that control the efficiency of LecA and StxB binding to Gb3: the structure of the fatty acyl chain of Gb3 (i.e. Gb3 species) and the order of the membrane environment. Both parameters determine how a single Gb3 molecule is embedded in the lipid bilayer, and hence, how the Gb3 head group is oriented. Thus, in accordance with previous studies^5,38,49^, we propose that the conformation of the carbohydrate head group of Gb3 is the critical factor that alters binding efficiencies for LecA and StxB. Importantly, we demonstrated here that the specific binding preferences of LecA and StxB are different. This enables utilizing the comparison of staining patterns of the two lectins as readout for the local membrane environment.

### The primary cilium harbors a unique lipid domain

The formation of distinct LecA and StxB domains on cells occurred on various scales. Even complete cellular organelles, like the primary cilium, were not recognized by LecA, while they were bound by StxB. The primary cilium membrane and the apical plasma membrane are continuous and there are no apparent obstacles that could directly block lipid movement along the outer plasma membrane leaflet^56^. Nevertheless, our surprising finding suggests that the primary cilium membrane contains a distinct lipid domain in which the head group of Gb3 is presented differently. This domain is of higher order and does not extend to the surrounding apical plasma membrane. Support for this hypothesis can be found in literature. Primary cilia were found to be cholesterol-rich^57,58^. The gangliosides GM1 and GM3^59^, and the Forssman glycolipid^60^, which are all considered as lipid raft markers, localize to the primary cilium of mammalian cells. Furthermore, *Trypanosoma brucei* flagellar membranes, which resemble primary cilia, are enriched in lipid raft components^61^.

### Influence of membrane order on LecA and StxB binding

It is well established that the lipid composition and overall membrane order differ between the apical and basolateral plasma membranes of epithelial cells^2,62,63^, with the apical plasma membrane displaying a higher order and containing more cholesterol and sphingolipids relative to the basolateral plasma membrane^64^. LecA and StxB were also able to recognize these differences, with StxB showing a significantly higher apical to basolateral signal ratio than LecA. This fits to the observations from our studies with artificial membranes, which revealed that, overall, LecA more tolerantly recognized different Gb3 species and membrane environments than StxB. By using GUVs, we were able to demonstrate in Fig. 4 that the apical preference of StxB can be explained by higher membrane order induced by cholesterol and sphingomyelin: For GUVs containing Gb3-mix, both LecA and StxB preferred the more ordered GUVs, but the preference was more pronounced for StxB, as it was for the apical to basolateral binding ratio on cells (Fig. 1A). When using GUVs containing only the single Gb3 species Gb3-C24:0, the effect became drastically enhanced. LecA bound equally to GUVs with and without cholesterol and sphingomyelin, whereas StxB only bound to GUVs with cholesterol and sphingomyelin.

It is known that StxB binding can subsequently influence the order of a lipid membrane because the toxin has binding sites for up to 15 Gb3 molecules^12^ and also drives its own endocytosis^8^. Nevertheless, the initial binding of StxB and LecA will be governed by the pre-existing accessibility of Gb3 headgroups and hence by the pre-existing order of the membrane. Accordingly, comparative StxB and LecA staining can be used to probe the pre-exisiting membrane order. This is verified by our finding that utilizing comparative StxB and LecA staining successfully replicated the previously known fact that apical plasma membranes of epithelial cells have a higher order in comparison to basolateral plasma membranes.

### Interplay between Gb3 species and membrane environment

Not only the total binding efficiencies of LecA and StxB at the apical and basolateral plasma membrane were different, but also on smaller scales distinct binding differences were seen: At the plasma membrane of energy depleted cells, LecA and StxB segregated into only partially overlapping nano-domains (Fig. 1C). This suggests that LecA and StxB segregation promptly develop after binding and most likely continue during endocytosis^9^, which provides an explanation for the different intracellular trafficking of LecA and StxB we reported before^9^. Importantly, we were able to rebuild such a domain separation between LecA and StxB in GUVs containing Gb3-mix and also in GUVs containing only the two Gb3 species Gb3-C24:0 and Gb3-FSL.

The reasons why Gb3-C24:0 and Gb3-FSL are minimally sufficient for reconstituting LecA and StxB segregation can be deduced from our experiments with single Gb3 species and illustrate how the interplay between Gb3 species and membrane environment governs different LecA and StxB binding: In phase-separated GUVs containing Gb3-FSL, both lectins preferred the ld phase over the lo phase. This was in stark contrast to the results obtained with phase-separated GUVs containing Gb3-mix and single non-FSL Gb3 species in which both lectins preferred the lo phase over the ld phase. Most likely, this is caused by a preferential integration of Gb3-FSL into the ld phase, since lectins bound to Gb3-FSL always co-localized with the ld marker β-BODIPY-FL-C5-HPC^17^ (Fig. S4B). As already described above, our GUV experiments with Gb3-C24:0 illustrate that for StxB Gb3 is best exposed in lo environments and the membrane composition alone is able to block or mask the binding of StxB.

The results from SLBs are in line with findings from GUVs in case of LecA, but for StxB divergent outcomes were observed: In phase-separated GUVs StxB hardly bound lo and ld phases, while on phase-separated SLBs binding of StxB to lo and ld phases occurred. This demonstrates that the system has a high sensitivity: Additional parameters that lead to subtle changes in the lipid bilayer and hence cause different Gb3 head group exposure, such as altered inter-leaflet coupling induced by the substrate of SLBs, can significantly shift the binding ability of StxB.

### Conclusion and outlook

Taken together, our data revealed that the two Gb3-binding lectins LecA and StxB segregate into different domains at the plasma membrane of cells. We were able to reconstitute these effects in synthetic membrane systems demonstrating that the driving force for segregation is controlled by parameters that determine the conformation of the Gb3 head group. These parameters, Gb3 fatty acyl chain structure and membrane environment, are interdependent, and enable LecA and StxB to act as sensors for membrane order on native cells with StxB indicating more ordered membranes. Thus, comparing LecA with StxB staining patterns could be used in future as a sensitive read out for determining membrane order during cellular processes.

## Material and Methods

### Cell lines and cell culture

Madin-Darby canine kidney (MDCK) strain II cells and HeLa cells were maintained at 37 °C and 5% CO_2_ in Dulbecco’s modified Eagle’s medium (DMEM) supplemented with 5% (for MDCK) and 10% (for HeLa) fetal calf serum. MDCK cells stably expressing Gb3 were established by stable overexpression of Gb3 synthase as described earlier^9,44^. For generating polarized monolayers, MDCK cells were confluently seeded to transwell filters (Corning, 0.4 µm pore size, polycarbonate membrane) and cultured for 4 d.

### Lipids and preparation of lipid mixtures

1,2-dioleoyl-sn-glycero-3-phosphocholine (DOPC), cholesterol (Chol) and natural porcine brain sphingomyelin (SM) were purchased from Avanti Polar Lipids, whereas Gb3-FSL was purchased from Sigma Aldrich. Gb3-mix was from Matreya, consisting of a mixture of Gb3 species extracted from red blood cells. The Gb3-species Gb3-C24:0 and Gb3-C24:1 were synthesized as described earlier^55^. The fluorescent lipid 2-(4,4-difluoro-5,7-dimethyl-4-bora-3a,4a-diaza-s-indacene-3-pentanoyl)-1-hexadecanoyl-sn-glycero-3-phosphocholine (β-BODIPY-FL-C5-HPC) was from Invitrogen. All lipids were dissolved in chloroform, only Gb3-mix and Gb3-species were dissolved in a 2:1 chloroform/methanol mixture. Dissolved lipids were stored with an argon layer on top at −20 °C. Lipid mixtures were prepared with a concentration of 0.5 mg of total lipid mass per 1 mL organic solvent or aqueous environment or buffers. The lipid ratios of each composition were calculated in molar percentages.

### Preparation of GUVs

GUVs were produced utilizing electro-swelling^65^. Briefly, 15 µl of the desired lipid mix with a concentration of 0.5 mg/ml was spread on each side of an indium-tin oxidized (ITO) glass slide (Präzisions Glas & Optik GmbH) with a Hamilton syringe (Hamilton) and evaporated overnight. Electro-swelling was done in water supplemented with 0.1 g/mL sucrose (Carl Roth) for 3 h at room temperature for non-phase-separated GUVs or for 3 h at 55 °C for phase-separated GUVs. The imaging buffer for GUVs was adjusted to the same osmolarity as the water/sucrose solution. Single GUVs were imaged after 20 min of lectin incubation. In case two lectins were investigated, the application was done simultaneously.

### Preparation of SLBs

Supported lipid membranes were prepared using vesicle fusion^66^. Briefly, lipid films were resuspended in low-Ca^2+^ buffer (20 mM TRIS/HCl, 100 mM NaCl, and 1 mM CaCl_2_, pH 7.5) at 55 °C for 35 min. Small vesicles were created using an extruder with a 50 nm cut-off polycarbonate membrane (Avestin) and directly transferred to the sample chamber. The sample chamber was home-built using mica, either grade V1 or V3 (Plano GmbH), which was fixed with optical adhesive (NOA 61, Norland Products) to a glass bottom dish (35 mm, #1.5 glass, World Precision Instruments). To induce vesicle fusion, a high-Ca^2+^ buffer (20 mM TRIS/HCl, 100 mM NaCl, and 10 mM CaCl_2_, pH 7.5) was added to increase the CaCl_2_ concentration and bilayers were heated to 55 °C for 1 h, followed by gentle cooling to room temperature. To remove non-fused vesicles and other debris, samples were rinsed with low-Ca^2+^ buffer. For microscopic imaging, SLBs were mounted and a time-series was recorded directly after addition of single lectins. In experiments with two lectins, StxB was applied first for 20 min and then LecA was applied.

### Lectin production and labeling

Recombinant LecA was produced from *Escherichia coli* following previously published procedures^39^. The StxB used was the B-subunit of Shiga toxin 1 produced in *Escherichia coli* (Sigma Aldrich). Lectins were dissolved at 1 mg/mL in ultrapure water (for StxB) or in PBS supplemented with 100 mg/L MgCl_2_ and 100 mg/L CaCl_2_ (for LecA), sterile filtered, and stored at 4 °C prior usage.

For fluorescent labeling, NHS-ester conjugated Alexa488 or Alexa647 (Thermo Fisher) and Cy3 or Cy5 (GE Healthcare Life Science) were used. Dyes were dissolved at a final concentration of 1 mg/mL in water-free DMSO (Carl Roth), aliquoted, and stored at −20 °C before usage. To set up the labeling reaction, 200 µL of lectin (1 mg/mL) was supplemented with 25 µL of a 1 M NaHCO_3_ (pH 9) solution. The molar ratio between dye and lectin was set to 5:1. The labeling mix was incubated at room temperature for 50 min and uncoupled dyes separated using Zeba Spin desalting columns (7k MWCO, 0.5 mL, Thermo Fischer). Labeled lectins were aliquoted into working stocks and stored at −80 °C until usage.

### Microscopy

MDCK cells grown on transwell filters were fixed with 4% formaldehyde and mounted as described before^9^.

GUVs and MDCK cell samples were imaged using a laser scanning confocal microscope system from Nikon (A1R), equipped with a 60× oil immersion objective with a numerical aperture (*NA*) of 1.49, and laser lines at 488 nm, 561 nm, and 640 nm. The following emission filters were used: for β-BODIPY-FL-C5-HPC and Alexa488: 525/50 BP filter, for Cy3: 595/50 BP filter, and for Cy5 and Alexa647: 700/75 BP filter. Image acquisition and processing was done using the software NIS-Elements (version 4.5, Nikon).

SLBs were imaged with a Nikon Eclipse Ti-E inverted microscope, equipped with a TIRF-illuminator, a 100× oil immersion objective (*NA* 1.49), with laser lines at 488 nm, 561 nm and 647 nm, and an EM-CCD camera (Andor Ixon DU-897). Images were acquired using a highly inclined laser beam (HILO-configuration)^67^. Image acquisition and processing was done using the software NIS-Elements (version 4.5, Nikon).

TIRF-SIM imaging was performed using a Nikon-N-SIM microscope equipped with laser lines at 488 nm and 561 nm, and a CFI apochromatic TIRF 100x oil immersion objective (*NA* 1.49).

### Image analysis

Quantification of lectin binding to MDCK cells was conducted by measuring fluorescent intensities in single cells that were not surrounded by labeled cells using a custom-written Matlab program as described before^68^.

Lectin binding efficiencies to GUVs were quantified using the ImageJ-macro GUV-AP^53^. Furthermore, we included a feature in the macro to enable the independent assessment of binding to ld and lo phases in phase-separated GUVs. To this end, we modified the macro with a procedure that recognizes and stores the locations of ld and lo domains from β-BODIPY-FL-C5-HPC staining patterns separately for all GUVs in a given image. The macro source code including the modifications is available at: https://github.com/AG-Roemer/GUV-AP/releases/tag/v2.0

### Energy depletion

HeLa cells were energy depleted by incubation for 20 min at 37 °C with PBS supplemented with 10 mM 2-deoxy-D-glucose (DOG) and 10 mM NaN_3_. The compounds were kept present during all incubation steps at 37 °C.

## Supplementary information

Supplementary Information.

## Data Availability

The datasets generated during and/or analysed during the current study are available from the corresponding authors on reasonable request.

## References

[1] van Meer G, Voelker DR, Feigenson GW (2008). Membrane lipids: where they are and how they behave. Nat. Rev. Mol. Cell Biol..

[2] Simons K, van Meer G (1988). Lipid sorting in epithelial cells. Biochemistry.

[3] Pomorski T, Hrafnsdóttir S, Devaux PF, van Meer G (2001). Lipid distribution and transport across cellular membranes. Semin. Cell developmental Biol..

[4] Sharom FJ (2011). Flipping and flopping–lipids on the move. IUBMB life.

[5] Lingwood D, Simons K (2010). Lipid rafts as a membrane-organizing principle. Science.

[6] Hancock JF (2006). Lipid rafts: contentious only from simplistic standpoints. Nat. Rev. Mol. Cell Biol..

[7] Munro S (2003). Lipid rafts: elusive or illusive?. Cell.

[8] Römer W (2007). Shiga toxin induces tubular membrane invaginations for its uptake into cells. Nature.

[9] Müller SK (2017). Gb3-binding lectins as potential carriers for transcellular drug delivery. Expert. Opin. drug. delivery.

[10] Chinnapen DJ-F (2012). Lipid sorting by ceramide structure from plasma membrane to ER for the cholera toxin receptor ganglioside GM1. Developmental Cell.

[11] Simons K, Toomre D (2000). Lipid rafts and signal transduction. Nat. Rev. Mol. Cell Biol..

[12] Windschiegl B (2009). Lipid Reorganization Induced by Shiga Toxin Clustering on Planar Membranes. PLoS ONE.

[13] Vereb G (2003). Dynamic, yet structured: The cell membrane three decades after the Singer-Nicolson model. Proc. Natl Acad. Sci. USA.

[14] Schubert T, Römer W (2015). How synthetic membrane systems contribute to the understanding of lipid-driven endocytosis. Biochimica et. biophysica acta.

[15] Pike LJ (2008). The challenge of lipid rafts. J. Lipid Res..

[16] Mayor S, Rao M (2004). Rafts: scale-dependent, active lipid organization at the cell surface. Traffic.

[17] Baumgart T, Hunt G, Farkas ER, Webb WW, Feigenson GW (2007). Fluorescence probe partitioning between Lo/Ld phases in lipid membranes. Biochimica et. biophysica acta.

[18] Sengupta P, Hammond A, Holowka D, Baird B (2008). Structural determinants for partitioning of lipids and proteins between coexisting fluid phases in giant plasma membrane vesicles. Biochimica et. biophysica acta.

[19] Klymchenko AS, Kreder R (2014). Fluorescent probes for lipid rafts: from model membranes to living cells. Chem. Biol..

[20] Sezgin E, Sadowski T, Simons K (2014). Measuring lipid packing of model and cellular membranes with environment sensitive probes. Langmuir: ACS J. Surf. colloids.

[21] Lingwood, C. A. Glycosphingolipid functions. *Cold Spring Harb Perspect Biol* **3**; 10.1101/cshperspect.a004788 (2011). 10.1101/cshperspect.a004788 PMC3119914 21555406

[22] Sezgin E (2012). Partitioning, diffusion, and ligand binding of raft lipid analogs in model and cellular plasma membranes. Biochim. Biophys. Acta.

[23] Baumgart T (2007). Large-scale fluid/fluid phase separation of proteins and lipids in giant plasma membrane vesicles. Proc. Natl Acad. Sci. U S Am..

[24] Blank N (2007). Cholera toxin binds to lipid rafts but has a limited specificity for ganglioside GM1. Immunology Cell Biol..

[25] Raghunathan K (2016). Glycolipid Crosslinking Is Required for Cholera Toxin to Partition Into and Stabilize Ordered Domains. Biophys. J..

[26] Dal Peraro M, van der Goot FG (2016). Pore-forming toxins. Ancient, but never really out of fashion. Nat. reviews. Microbiology.

[27] Cioci G (2003). Structural basis of calcium and galactose recognition by the lectin PA-IL of Pseudomonas aeruginosa. FEBS Lett..

[28] Kirkeby S, Hansen AK, d’Apice A, Moe D (2006). The galactophilic lectin (PA-IL, gene LecA) from Pseudomonas aeruginosa. Its binding requirements and the localization of lectin receptors in various mouse tissues. Microb. pathogenesis.

[29] Chen CP, Song SC, Gilboa-Garber N, Chang KS, Wu AM (1998). Studies on the binding site of the galactose-specific agglutinin PA-IL from Pseudomonas aeruginosa. Glycobiology.

[30] Pina DG, Johannes L (2005). Cholera and Shiga toxin B-subunits: thermodynamic and structural considerations for function and biomedical applications. Toxicon.

[31] Pina DG, Johannes L, Castanho, Miguel ARB (2007). Shiga toxin B-subunit sequential binding to its natural receptor in lipid membranes. Biochim. Biophys. Acta.

[32] St. Hilaire, Phaedria M, Boyd MK, Toone EJ (1994). Interaction of the Shiga-like Toxin Type 1 B-Subunit with Its Carbohydrate Receptor. Biochemistry.

[33] Johannes L, Römer W (2010). Shiga toxins–from cell biology to biomedical applications. Nat. Rev. Microbiol..

[34] Dietrich C (2001). Lipid Rafts Reconstituted in Model Membranes. Biophysical J..

[35] Sych, T., Mély, Y. & Römer, W. Lipid self-assembly and lectin-induced reorganization of the plasma membrane. *Philosophical transactions of the Royal Society of London. Series B, Biological sciences* **373**; 10.1098/rstb.2017.0117 (2018). 10.1098/rstb.2017.0117 PMC5904303 29632269

[36] Simons K, Sampaio JL (2011). Membrane organization and lipid rafts. Cold Spring Harb. Perspect. Biol..

[37] Simons K, Gerl MJ (2010). Revitalizing membrane rafts: new tools and insights. Nat. Rev. Mol. Cell Biol..

[38] Lingwood D (2011). Cholesterol modulates glycolipid conformation and receptor activity. Nat. Chem. Biol..

[39] Blanchard B (2008). Structural Basis of the Preferential Binding for Globo-Series Glycosphingolipids Displayed by Pseudomonas aeruginosa Lectin I. J. Mol. Biol..

[40] Worstell NC (2018). Hetero-Multivalency of Pseudomonas aeruginosa Lectin LecA Binding to Model Membranes. Sci. Rep..

[41] Stroukov W (2019). Synchronizing Protein Traffic to the Primary Cilium. Front. Genet..

[42] Jacewicz M (1986). Pathogenesis of shigella diarrhea. XI. Isolation of a shigella toxin- binding glycolipid from rabbit jejunum and HeLa cells and its identification as globotriaosylceramide. J. Exp. Med..

[43] Villringer S (2018). Lectin-mediated protocell crosslinking to mimic cell-cell junctions and adhesion. Sci. Rep..

[44] Eierhoff T (2014). A lipid zipper triggers bacterial invasion. Proc. Natl Acad. Sci. U S Am..

[45] Arab S, Lingwood CA (1996). Influence of phospholipid chain length on verotoxin/globotriaosyl ceramide binding in model membranes: comparison of a supported bilayer film and liposomes. Glycoconj. J..

[46] Binnington B, Lingwood D, Nutikka A, Lingwood CA (2002). Effect of globotriaosyl ceramide fatty acid alpha-hydroxylation on the binding by verotoxin 1 and verotoxin 2. Neurochem. Res..

[47] Kiarash A, Boyd B, Lingwood CA (1994). Glycosphingolipid receptor function is modified by fatty acid content. Verotoxin 1 and verotoxin 2c preferentially recognize different globotriaosyl ceramide fatty acid homologues. J. Biol. Chem..

[48] Lingwood C (1996). Role of verotoxin receptors in pathogenesis. Trends Microbiology.

[49] Lingwood CA (2010). New aspects of the regulation of glycosphingolipid receptor function. Chem. Phys. Lipids.

[50] Pellizzari A, Pang H, Lingwood CA (1992). Binding of verocytotoxin 1 to its receptor is influenced by differences in receptor fatty acid content. Biochemistry.

[51] Lingwood CA, Binnington B, Manis A, Branch DR (2010). Globotriaosyl ceramide receptor function - where membrane structure and pathology intersect. FEBS Lett..

[52] Blake, D. A., Bovin, N. V., Bess, D. & Henry, S. M. FSL constructs: a simple method for modifying cell/virion surfaces with a range of biological markers without affecting their viability. *Journal of visualized experiments: JoVE*; 10.3791/3289 (2011). 10.3791/3289 PMC3211133 21847082

[53] Sych, T. *et al*. GUV-AP: multifunctional FIJI-based tool for quantitative image analysis of Giant Unilamellar Vesicles. *Bioinformatics (Oxford, England)*; 10.1093/bioinformatics/bty962 (2018). 10.1093/bioinformatics/bty962 30475993

[54] Simons K, Vaz, Winchil LC (2004). Model systems, lipid rafts, and cell membranes. Annu. Rev. Biophys. Biomol. Struct..

[55] Schütte OM (2014). Influence of Gb3 glycosphingolipids differing in their fatty acid chain on the phase behaviour of solid supported membranes: chemical syntheses and impact of Shiga toxin binding. Chem. Sci..

[56] Reiter JF, Blacque OE, Leroux MR (2012). The base of the cilium: roles for transition fibres and the transition zone in ciliary formation, maintenance and compartmentalization. EMBO Rep..

[57] Ott, C. & Lippincott-Schwartz, J. Visualization of live primary cilia dynamics using fluorescence microscopy. *Current protocols in cell biology* **Chapter 4**, Unit 4.26; 10.1002/0471143030.cb0426s57 (2012). 10.1002/0471143030.cb0426s57 PMC3690948 23208547

[58] Cuevas P, Gutierrez Diaz JA (1985). Absence of filipin-sterol complexes from the ciliary necklace of ependymal cells. Anat. embryology.

[59] Janich P, Corbeil D (2007). GM1 and GM3 gangliosides highlight distinct lipid microdomains within the apical domain of epithelial cells. FEBS Lett..

[60] Vieira OV (2006). FAPP2, cilium formation, and compartmentalization of the apical membrane in polarized Madin-Darby canine kidney (MDCK) cells. Proc. Natl Acad. Sci. USA.

[61] Tyler KM (2009). Flagellar membrane localization via association with lipid rafts. J. Cell Sci..

[62] Thuenauer R (2011). A PDMS-based biochip with integrated sub-micrometre position control for TIRF microscopy of the apical cell membrane. Lab. a chip.

[63] Owen DM, Magenau A, Majumdar A, Gaus K (2010). Imaging membrane lipid order in whole, living vertebrate organisms. Biophys. J..

[64] Maxfield FR, Wüstner D (2002). Intracellular cholesterol transport. J. Clin. investigation.

[65] Madl, J., Villringer, S. & Römer, W. in *Chemical and synthetic approaches in membrane biology*, edited by A. K. Shukla (Humana Press, New York, NY, 2017), pp. 17–36.

[66] Tamm LK, McConnell HM (1985). Supported phospholipid bilayers. Biophysical J..

[67] Tokunaga M, Imamoto N, Sakata-Sogawa K (2008). Highly inclined thin illumination enables clear single-molecule imaging in cells. Nat. methods.

[68] Thuenauer R (2014). Four-dimensional live imaging of apical biosynthetic trafficking reveals a post-Golgi sorting role of apical endosomal intermediates. Proc. Natl Acad. Sci. USA.

