# Supplementary material for: Differential recognition of lipid domains by two Gb3-binding lectins
Source: Sci Rep. 2020 Jun 16;10:9752. doi: 10.1038/s41598-020-66522-8 (PMC7297801; doi:10.1038/s41598-020-66522-8)
Supplement: Supplementary file 1 — Supplementary Information. [file 41598_2020_66522_MOESM1_ESM.pdf]

Supplementary Information

Differential recognition of lipid domains by two Gb3-binding lectins

Thomas Schubert[a,b,c], Taras Sych[a,b,d], Josef Madl[a,b], Maokai Xu[a,b], Ramin Omidvar[a,b], Lukas J. Patalag[e], Annika Ries[f], Katharina Kettelhoit[e], Annette Brandel[a,b], Yves Mely[d], Claudia Steinem[f], Daniel B. Werz[e], Roland Thuenauer[a,b,g,1], Winfried Römer[a,b,1]

[a] Faculty of Biology, Albert-Ludwigs-University Freiburg, Freiburg, Germany

[b] Synthetic Biology of Signalling Processes, Signalling Research Centres BIOSS and CIBSS, Albert-Ludwigs-University Freiburg, Freiburg, Germany

[c] Toolbox, BIOSS Centre for Biological Signalling Studies, Albert-Ludwigs-University Freiburg, Freiburg, Germany

[d] Laboratory of Bioimaging and Pathologies, UMR 7021 CNRS, Faculty of Pharmacy, University of Strasbourg, France

[e] Institut für Organische Chemie, TU Braunschweig, Braunschweig, Germany

[f] Institut für Organische und Biomolekulare Chemie, Georg-August-Universität Göttingen, Göttingen, Germany.

[g] Advanced Light and Fluorescence Microscopy Facility, Centre for Structural Systems Biology (CSSB) and University of Hamburg, Hamburg, Germany

[1] To whom correspondence should be addressed. Email: roland.thuenauer@cssb-hamburg.de (RT), winfried.roemer@bioss.uni-freiburg.de (WR)

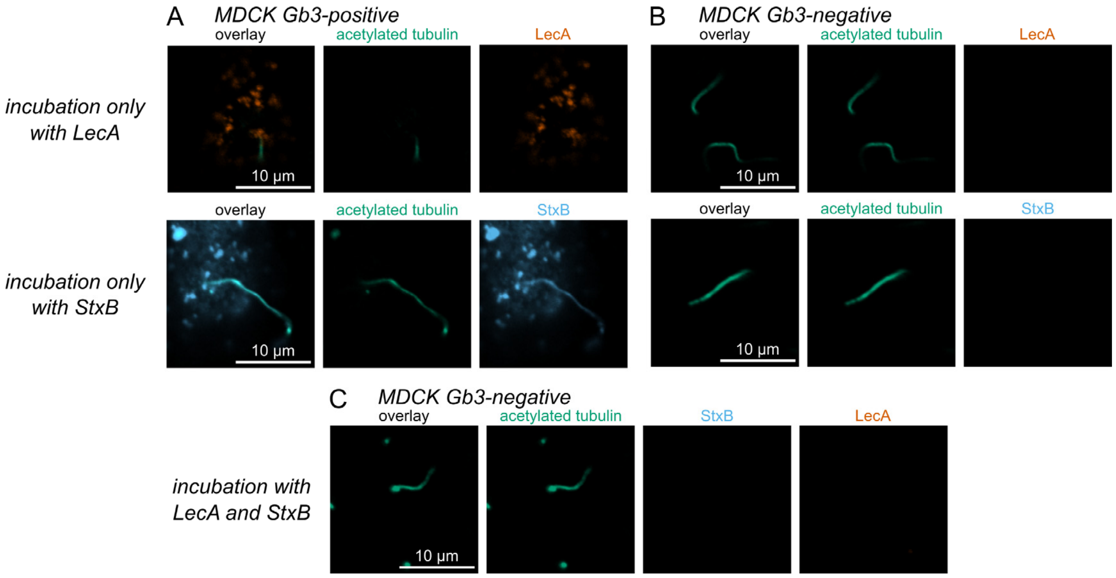

Figure S1: Control experiments with Gb3-positive and Gb3-negative MDCK cells

Polarized MDCK cells either stably expressing Gb3 synthase (MDCK Gb3-positive, (A)) or not (MDCK Gb3-negative, (B) and (C)) were incubated apically with LecA-Alexa488 (196 nM, orange) and/or StxB-Cy3 (13 nM, blue) as indicated for 30 min at 37°C. After washout of unbound lectin, cells were fixed and primary cilia were stained using an antibody recognizing acetylated tubulin (green). The images show confocal sections at the height of the apical plasma membrane.

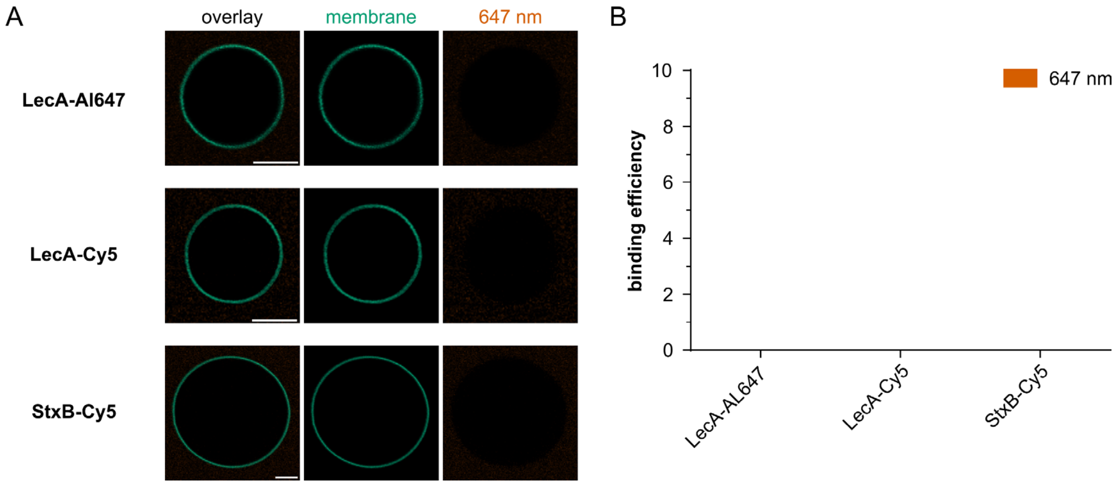

Figure S2: GUVs lacking Gb3 do not bind LecA or StxB

(A) GUVs composed of DOPC/cholesterol/β-BODIPY-FL-C_5-HPC (green) in the ratio 69.5/30/0.5 mol-% were incubated with 200 nM of either LecA-Al647, or LecA-Cy5, or with StxB-Cy5 (orange). The depicted representative equatorial cross sections were acquired with identical microscope settings as for the quantitative binding studies. Scale bars correspond to 5 µm. (B) Quantification of the binding efficiencies, showing that no lectin binding occurs in GUVs without Gb3.

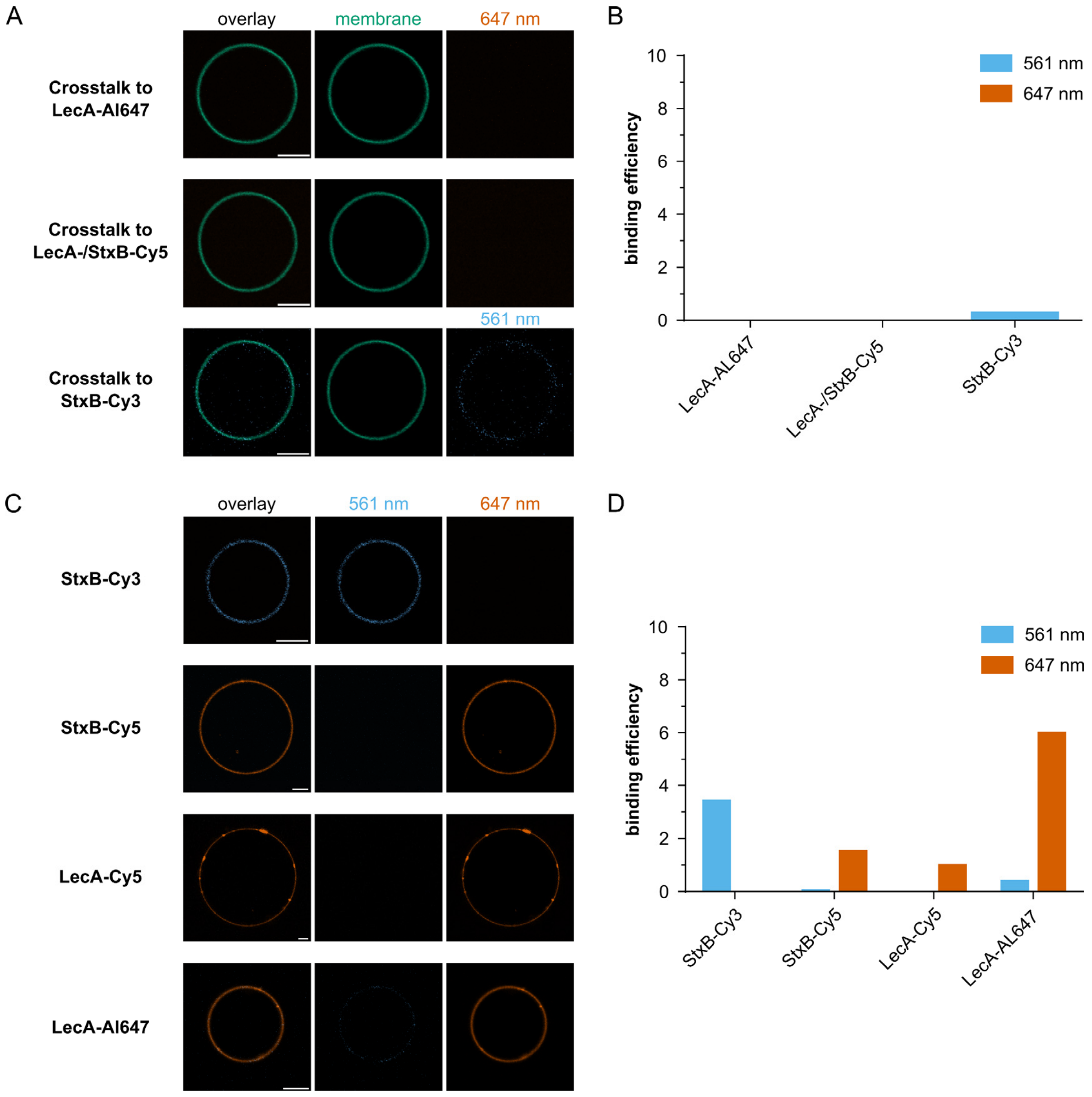

Figure S3: Controls for spectral crosstalk

(A) GUVs composed of DOPC/cholesterol/β-BODIPY-FL-C_5-HPC (green)/Gb3-mix in the ratio 64.5/30/0.5/5 mol-% were imaged using the same microscope settings as for the quantitative binding studies. Since no lectins were added, signals in the images recorded at 647 nm excitation (orange, upper and middle row) represent crosstalk signals from the membrane channel to the channel normally used for LecA-Al647, LecA-Cy5, and StxB-Cy5 imaging, whereas signals in the images at 561 nm excitation (blue, lower row) represent crosstalk signals from the membrane channel to the channel normally used for StxB-Cy3 imaging. Scale bars correspond to 5 µm. (B) Quantification of the (apparent) binding efficiencies for the GUVs displayed in (A) as estimate of the spectral crosstalk. (C) GUVs consisting of DOPC/cholesterol/Gb3-mix in the ratio 65/30/5 mol-% were

incubated with 200 nM of either StxB-Cy3, StxB-Cy5, LecA-Cy5, or LecA-Al647 and were imaged using the same microscope settings as for the quantitative binding studies. For GUVs incubated with StxB-Cy3 (first row), the signal from the lectin was measured using 561 nm excitation (blue) and also spectral crosstalk signals were measured using 647 nm excitation (orange). For GUVs incubated with StxB-Cy5, LecA-Cy5, and LecA-Al647 (rows two - four), the signal from the lectins was measured using 647 nm excitation (orange) and spectral crosstalk signals were measured using 561 nm excitation (blue). Scale bars correspond to 5 µm. (D) Quantification of the binding efficiencies for the GUVs displayed in (C). For StxB-Cy3, the 561 nm - signal represents a true lectin binding efficiency, whereas the 647 nm – signal is an apparent binding efficiency representing the spectral crosstalk into this channel. For StxB-Cy5, LecA-Cy5, and LecA-Al647, it is opposite: the 561 nm - signal represents crosstalk and the 647 nm - signal represents lectin binding efficiency.

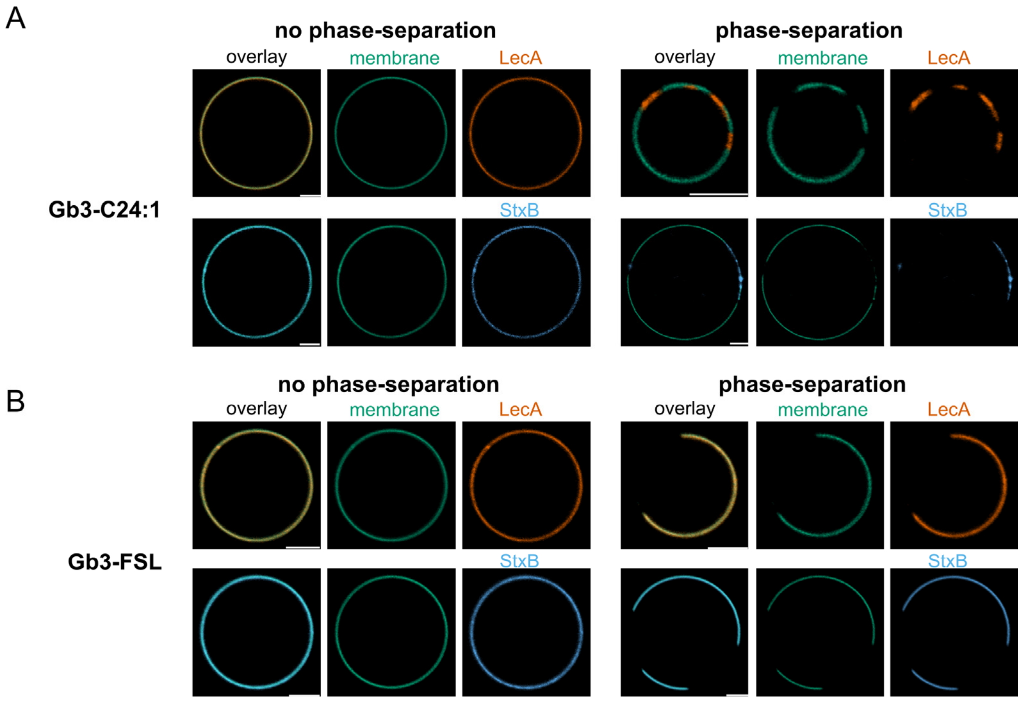

Figure S4: Additional data related to Figure 3

GUVs doped with 0.5 mol-% of the membrane marker β-BODIPY-FL-C_5-HPC (green) and 5 mol-% Gb3-C24:1 (A), or 5 mol-% Gb3-FSL (B) were incubated with LecA-Cy5 (200 nM, orange) or StxB-Cy5 (200 nM, blue). Non-phase-separated GUVs (left panels) contained DOPC/cholesterol/Gb3 in the ratio 64.5/30/5 mol-%, whereas phase-separated GUVs (right panels) consisted of DOPC/cholesterol/sphingomyelin/Gb3 in the ratio 42.5/14.5/42.5/5 mol-%. Scale bars correspond to 5 µm.

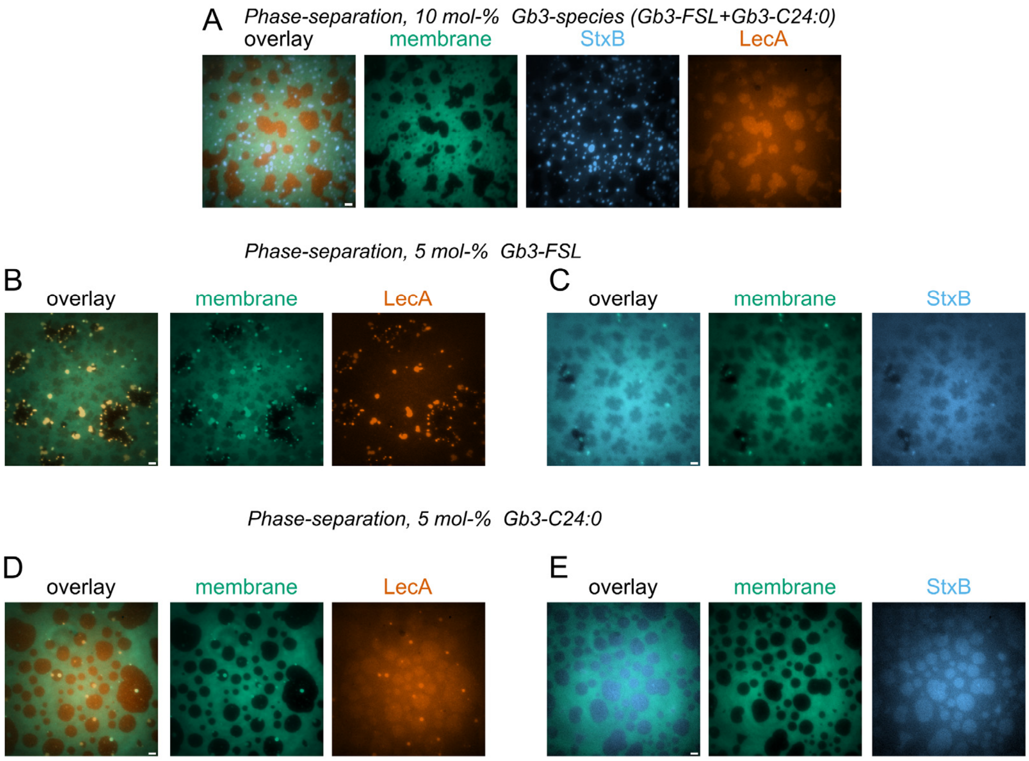

Figure S5: Investigating LecA and StxB binding on SLBs containing Gb3-FSL and/or Gb3-C24:0

(A) Phase-separated SLBs consisting of DOPC/cholesterol/sphingomyelin/β-BODIPY-FL-C_5-HPC (green)/Gb3-FSL/Gb3-C24:0 in the ratio 37.9/14.1/37.9/0.1/5/5 mol-%) were incubated with StxB-Cy3 (100 nM, blue) and LecA-Al647 (100 nM, orange). (B-E) Phase-separated SLBs consisting of DOPC/cholesterol/sphingomyelin/β-BODIPY-FL-C_5-HPC (green) in the ratio 37.4/20/37.5/0.1 mol-% and either 5 mol-% of Gb3-FSL (B, C) or 5 mol-% of Gb3-C24:0 (D, E) were incubated with either 200 nM LecA-Cy5 (200 nM, orange, C and D) or StxB-Cy5 (200 nM, blue, D and E). Scale bars correspond to 5 µm.

Table S1: Descriptive statistics of the data displayed in Figs. 3E-F

	**Minimum**		**Maximum**		**Mean**		**Std. Deviation**		**Single GUVs measured**	
	LecA	StxB	LecA	StxB	LecA	StxB	LecA	StxB	LecA	StxB
**1** Gb3-mix No Phase	0.443	0.436	2.487	2.020	1.279	1.095	0.586	0.336	36	39
**2** Gb3-mix Phase Ld	0.169	0	2.349	0.329	0.921	0.054	0.517	0.096	33	27
**3** Gb3-mix Phase Lo	3.044	1.722	9.446	7.038	6.570	4.520	2.007	1.452	33	27
**4** Gb3-FSL No Phase	1.450	2.226	4.368	7.192	2.822	4.505	0.837	1.287	37	46
**5** Gb3-FSL Phase Ld	1.356	2.769	7.471	7.128	4.144	4.317	1.600	0.915	17	22
**6** Gb3-FSL Phase Lo	0.041	0.125	1.685	1.069	0.728	0.619	0.464	0.251	17	22
**7** Gb3-C24:0 No Phase	0.103	0.172	3.758	1.744	1.089	0.742	1.019	0.460	44	41
**8** Gb3-C24:0 Phase Ld	0	0	0.798	0	0.106	0	0.171	0	41	51
**9** Gb3-C24:0 Phase Lo	0.525	0	3.819	0.370	1.728	0.045	0.909	0.077	41	51
**10** Gb3-C24:1 No Phase	0.810	0.288	3.203	3.345	1.671	1.400	0.586	0.915	50	39
**11** Gb3-C24:1 Phase Ld	0.338	0	2.862	1.203	1.110	0.334	0.563	0.285	35	27
**12** Gb3-C24:1 Phase Lo	1.408	0.425	6.770	5.473	3.777	2.448	1.300	1.060	35	27

Table S2: Statistical analysis of the data from Figs. 3E-F

Statistical analysis was carried out with GraphPad Prism (Version 6.0) with a two-way ANOVA Tukey´s multiple comparisons test. The significance level was set to 0.05.

		LecA			StxB		
**Condition (I)**	**Condition (J)**	Mean Difference (I-J)	95% CI of difference	Significance	Mean Difference (I-J)	95% CI of difference	Significance
**1** Gb3-mix No Phase	**2** Gb3-mix Phase Ld	0.357	-0,4111 to 1,126	No	1.041	0,4296 to 1,653	Yes****
	**3** Gb3-mix Phase Lo	-5.291	-6,059 to -4,523	Yes****	-3.425	-4,037 to -2,814	Yes****
	**4** Gb3-FSL No Phase	-1.544	-2,290 to -0,7973	Yes****	-3.411	-3,942 to -2,879	Yes****
	**5** Gb3-FSL Phase Ld	-2.865	-3,803 to -1,927	Yes****	-3.222	-3,874 to -2,571	Yes****
	**6** Gb3-FSL Phase Lo	0.550	-0,3879 to 1,489	No	0.476	-0,1751 to 1,127	No
	**7** Gb3-C24:0 No Phase	0.190	-0,5266 to 0,9066	No	0.353	-0,1936 to 0,8990	No
	**8** Gb3-C24:0 Phase Ld	1.173	0,4444 to 1,901	Yes****	1.095	0,5753 to 1,614	Yes****
	**9** Gb3-C24:0 Phase Lo	-0.450	-1,178 to 0,2785	No	1.050	0,5302 to 1,569	Yes****
	**10** Gb3-C24:1 No Phase	-0.393	-1,090 to 0,3043	No	-0.305	-0,8579 to 0,2483	No
	**11** Gb3-C24:1 Phase Ld	0.169	-0,5882 to 0,9256	No	0.761	0,1493 to 1,372	Yes**
	**12** Gb3-C24:1 Phase Lo	-2.498	-3,255 to -1,742	Yes****	-1.353	-1,965 to -0,7420	Yes****
**2** Gb3-mix Phase Ld	**3** Gb3-mix Phase Lo	-5.648	-6,433 to -4,863	Yes****	-4.466	-5,131 to -3,802	Yes****
	**4** Gb3-FSL No Phase	-1.901	-2,665 to -1,138	Yes****	-4.452	-5,044 to -3,860	Yes****
	**5** Gb3-FSL Phase Ld	-3.222	-4,174 to -2,270	Yes****	-4.263	-4,965 to -3,562	Yes****
	**6** Gb3-FSL Phase Lo	0.193	-0,7589 to 1,145	No	-0.565	-1,266 to 0,1365	No
	**7** Gb3-C24:0 No Phase	-0.167	-0,9017 to 0,5669	No	-0.688	-1,294 to -0,08309	Yes*
	**8** Gb3-C24:0 Phase Ld	0.815	0,06957 to 1,561	Yes*	0.054	-0,5276 to 0,6350	No
	**9** Gb3-C24:0 Phase Lo	-0.807	-1,553 to -0,06141	Yes*	0.009	-0,5726 to 0,5899	No
	**10** Gb3-C24:1 No Phase	-0.750	-1,465 to -0,03490	Yes*	-1.346	-1,957 to -0,7344	Yes****
	**11** Gb3-C24:1 Phase Ld	-0.189	-0,9624 to 0,5850	No	-0.280	-0,9451 to 0,3844	No
	**12** Gb3-C24:1 Phase Lo	-2.856	-3,630 to -2,082	Yes****	-2.395	-3,059 to -1,730	Yes****

**3** Gb3-mix Phase Lo	**4** Gb3-FSL No Phase	3.747	2,984 to 4,511	Yes****	0.015	-0,5775 to 0,6068	No
	**5** Gb3-FSL Phase Ld	2.426	1,474 to 3,378	Yes****	0.203	-0,4986 to 0,9043	No
	**6** Gb3-FSL Phase Lo	5.841	4,889 to 6,793	Yes****	3.901	3,200 to 4,603	Yes****
	**7** Gb3-C24:0 No Phase	5.481	4,747 to 6,215	Yes****	3.778	3,173 to 4,383	Yes****
	**8** Gb3-C24:0 Phase Ld	6.464	5,718 to 7,209	Yes****	4.520	3,939 to 5,101	Yes****
	**9** Gb3-C24:0 Phase Lo	4.841	4,096 to 5,587	Yes****	4.475	3,894 to 5,056	Yes****
	**10** Gb3-C24:1 No Phase	4.898	4,183 to 5,613	Yes****	3.120	2,509 to 3,732	Yes****
	**11** Gb3-C24:1 Phase Ld	5.460	4,686 to 6,233	Yes****	4.186	3,521 to 4,851	Yes****
	**12** Gb3-C24:1 Phase Lo	2.793	2,019 to 3,566	Yes****	2.072	1,407 to 2,736	Yes****
**4** Gb3-FSL No Phase	**5** Gb3-FSL Phase Ld	-1.321	-2,255 to -0,3869	Yes***	0.188	-0,4449 to 0,8213	No
	**6** Gb3-FSL Phase Lo	2.094	1,160 to 3,028	Yes****	3.887	3,254 to 4,520	Yes****
	**7** Gb3-C24:0 No Phase	1.734	1,023 to 2,445	Yes****	3.763	3,239 to 4,288	Yes****
	**8** Gb3-C24:0 Phase Ld	2.716	1,993 to 3,439	Yes****	4.505	4,009 to 5,002	Yes****
	**9** Gb3-C24:0 Phase Lo	1.094	0,3710 to 1,817	Yes****	4.460	3,964 to 4,957	Yes****
	**10** Gb3-C24:1 No Phase	1.151	0,4596 to 1,843	Yes****	3.106	2,574 to 3,637	Yes****
	**11** Gb3-C24:1 Phase Ld	1.712	0,9606 to 2,464	Yes****	4.171	3,579 to 4,763	Yes****
	**12** Gb3-C24:1 Phase Lo	-0.955	-1,707 to -0,2028	Yes**	2.057	1,465 to 2,649	Yes****
**5** Gb3-FSL Phase Ld	**6** Gb3-FSL Phase Lo	3.415	2,322 to 4,509	Yes****	3.698	2,962 to 4,435	Yes****
	**7** Gb3-C24:0 No Phase	3.055	2,144 to 3,966	Yes****	3.575	2,930 to 4,221	Yes****
	**8** Gb3-C24:0 Phase Ld	4.038	3,118 to 4,957	Yes****	4.317	3,694 to 4,940	Yes****
	**9** Gb3-C24:0 Phase Lo	2.415	1,495 to 3,335	Yes****	4.272	3,649 to 4,895	Yes****
	**10** Gb3-C24:1 No Phase	2.472	1,577 to 3,368	Yes****	2.918	2,266 to 3,569	Yes****
	**11** Gb3-C24:1 Phase Ld	3.034	2,091 to 3,976	Yes****	3.983	3,282 to 4,685	Yes****
	**12** Gb3-C24:1 Phase Lo	0.367	-0,5761 to 1,309	No	1.869	1,167 to 2,570	Yes****
**6** Gb3-FSL Phase Lo	**7** Gb3-C24:0 No Phase	-0.361	-1,271 to 0,5501	No	-0.123	-0,7689 to 0,5220	No
	**8** Gb3-C24:0 Phase Ld	0.622	-0,2976 to 1,542	No	0.619	-0,004255 to 1,242	No
	**9** Gb3-C24:0 Phase Lo	-1.000	-1,920 to -0,08038	Yes*	0.574	-0,04934 to 1,197	No

	**10** Gb3-C24:1 No Phase	-0.943	-1,838 to -0,04791	Yes*	-0.781	-1,432 to -0,1297	Yes**
	**11** Gb3-C24:1 Phase Ld	-0.382	-1,324 to 0,5609	No	0.285	-0,4168 to 0,9861	No
	**12** Gb3-C24:1 Phase Lo	-3.049	-3,992 to -2,106	Yes****	-1.830	-2,531 to -1,128	Yes****
**7** Gb3-C24:0 No Phase	**8** Gb3-C24:0 Phase Ld	0.983	0,2905 to 1,675	Yes***	0.742	0,2298 to 1,254	Yes***
	**9** Gb3-C24:0 Phase Lo	-0.640	-1,332 to 0,05240	No	0.697	0,1848 to 1,209	Yes***
	**10** Gb3-C24:1 No Phase	-0.583	-1,242 to 0,07644	No	-0.658	-1,204 to -0,1112	Yes**
	**11** Gb3-C24:1 Phase Ld	-0.021	-0,7435 to 0,7009	No	0.408	-0,1973 to 1,013	No
	**12** Gb3-C24:1 Phase Lo	-2.688	-3,411 to -1,966	Yes****	-1.706	-2,311 to -1,101	Yes****
**8** Gb3-C24:0 Phase Ld	**9** Gb3-C24:0 Phase Lo	-1.622	-2,327 to -0,9182	Yes****	-0.045	-0,5287 to 0,4386	No
	**10** Gb3-C24:1 No Phase	-1.565	-2,237 to -0,8935	Yes****	-1.400	-1,919 to -0,8801	Yes****
	**11** Gb3-C24:1 Phase Ld	-1.004	-1,738 to -0,2702	Yes***	-0.334	-0,9154 to 0,2472	No
	**12** Gb3-C24:1 Phase Lo	-3.671	-4,405 to -2,937	Yes****	-2.448	-3,030 to -1,867	Yes****
**9** Gb3-C24:0 Phase Lo	**10** Gb3-C24:1 No Phase	0.057	-0,6147 to 0,7289	No	-1.355	-1,874 to -0,8350	Yes****
	**11** Gb3-C24:1 Phase Ld	0.618	-0,1154 to 1,352	No	-0.289	-0,8703 to 0,2923	No
	**12** Gb3-C24:1 Phase Lo	-2.049	-2,783 to -1,315	Yes****	-2.403	-2,985 to -1,822	Yes****
**10** Gb3-C24:1 No Phase	**11** Gb3-C24:1 Phase Ld	0.561	-0,1414 to 1,264	No	1.066	0,4541 to 1,677	Yes****
	**12** Gb3-C24:1 Phase Lo	-2.106	-2,809 to -1,403	Yes****	-1.049	-1,660 to -0,4372	Yes****
**11** Gb3-C24:1 Phase Ld	**12** Gb3-C24:1 Phase Lo	-2.667	-3,429 to -1,905	Yes****	-2.114	-2,779 to -1,449	Yes****

Table S3: Descriptive statistics of the data displayed in Figs. 4E-F

	**Minimum**		**Maximum**		**Mean**		**Std. Deviation**		**Single GUVs measured**	
	LecA	StxB	LecA	StxB	LecA	StxB	LecA	StxB	LecA	StxB
**1** Gb3-mix + DOPC	2.478	0.064	7.385	0.462	4.205	0.296	1.329	0.074	13	38
**2** Gb3-mix + Chol + SM	3.445	0.358	8.309	2.345	5.774	1.104	1.311	0.370	27	39
**3** Gb3-C24:0 + DOPC	0.666	0	2.561	0	1.436	0	0.423	0	39	36
**4** Gb3-C24:0 + Chol + SM	0	0	4.451	0.674	1.692	0.142	1.044	0.135	36	51

Table S4: Statistical analysis of the data from Figs. 4E-F

Statistical analysis was carried out with GraphPad Prism (Version 6.0) with a two-way ANOVA Tukey´s multiple comparisons test. The significance level was set to 0.05.

		**LecA**			**StxB**		
**Condition (I)**	**Condition (J)**	Mean Difference (I-J)	95% CI of difference	Significance	Mean Difference (I-J)	95% CI of difference	Significance
**1** Gb3-mix + DOPC	**2** Gb3-mix + Chol + SM	-1.568	-2.448 to -0.6887	Yes****	-0.808	-0.9251 to -0.6902	Yes****
	**3** Gb3-C24:0 + DOPC	2.770	1.935 to 3.605	Yes****	0.296	0.1760 to 0.4157	Yes****
	**4** Gb3-C24:0 + Chol + SM	2.513	1.670 to 3.357	Yes****	0.154	0.04350 to 0.2644	Yes**
**2** Gb3-mix + Chol + SM	**3** Gb3-C24:0 + DOPC	4.338	3.686 to 4.991	Yes****	1.104	0.9844 to 1.223	Yes****
	**4** Gb3-C24:0 + Chol + SM	4.082	3.418 to 4.745	Yes****	0.962	0.8520 to 1.071	Yes****
**3** Gb3-C24:0 + DOPC	**4** Gb3-C24:0 + Chol + SM	-0.256	-0.8588 to 0.3459	No	-0.142	-0.2541 to -0.02976	Yes**
